# Selecting Stable Metal Oxide Nanoparticle Additives for Hydrocarbon Fuels through a Predictive Thermal Stability Phase Map

**DOI:** 10.1021/acsomega.6c05279

**Published:** 2026-06-30

**Authors:** Mehmet Selman Gökmen

**Affiliations:** Seydişehir Vocational High School, Department of Motor Vehicles and Transportation Technologies, Necmettin Erbakan University, Konya 42090, Türkiye

## Abstract

Metal oxide nanoparticles are widely investigated as fuel additives in internal combustion engines, where they can enhance combustion efficiency and reduce emissions; however, their practical use is fundamentally constrained by the colloidal instability of the resulting nanofuels, triggered by the drastic reduction in base fluid viscosity at elevated temperatures. This study presents a predictive framework that delineates the thermophysical kinetic stability boundaries for ten different metal oxide nanoparticles in hydrocarbon media (iso-octane and n-dodecane, representing gasoline and diesel surrogates), independent of chemical stabilization. While the analytical model covers a broad range of oxides, the theoretical stability boundaries were experimentally corroborated as an idealized baseline through dynamic light scattering (DLS) measurements using three representative nanoparticles (CeO_2_, Fe_2_O_3_, SiO_2_) selected to span the entire density spectrum. Analytical modeling based on a Lagrangian perspective, conducted within the 20–100 °C range, revealed that a temperature-induced viscosity loss of 55–65% suppresses the gain in thermal energy, driving the system into an advective sedimentation regime. Stability phase maps, constructed using the Peclet number and critical settling velocities, characterize safe operational domains as a function of particle density and temperature. The experimental results corroborated the theoretical limits, specifically showing that high-density CeO_2_ undergoes catastrophic agglomeration (>600 nm) in iso-octane at 80 °C due to viscosity collapse. This study establishes a theoretical stability ceiling for nanofuel design and provides a transferable, transport-physics-based methodology for selecting a stable combination of nanoparticle type, base fuel, and operating temperature, transforming the conventional experimental trial-and-error process of nanofuel formulation into a rational design workflow.

## Graphical abstract

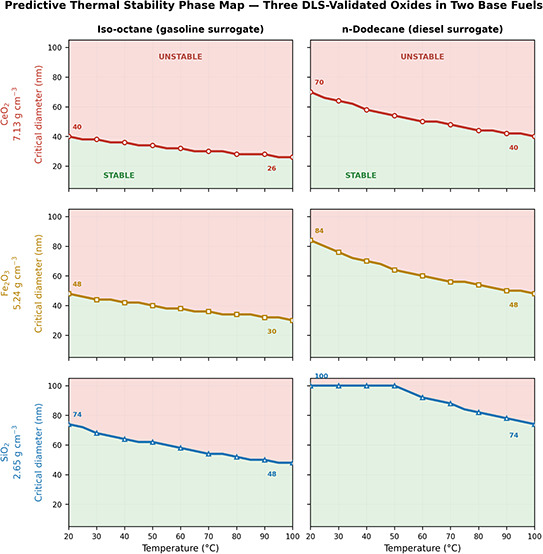

## 1 Introduction

Metal oxide-based nanofuels are being investigated with increasing momentum in the literature, owing to their superior thermal conductivity properties that enhance heat transfer and combustion performance compared to conventional base fluids. In particular, the dispersion of representative catalytic metal oxides (e.g., cerium oxide , aluminum oxide , titanium dioxide , copper oxide) into hydrocarbon fuels such as iso-octane and n-dodecane holds the potential to increase combustion efficiency, reduce ignition delay, and mitigate soot emissions in internal combustion engines and propulsion systems. This interest spans both conventional fossil fuels and alternative fuels such as biodiesel and its blends, where nanoparticle additives have shown particular promise. Recent experimental studies have quantified these benefits; for instance, Kumbhar et al.^1^ reported that CeO_2_ and Al_2_O_3_ nanoparticles increased brake thermal efficiency by 17–18% compared to conventional fuel, while^2^ observed improvements in heat release rates ranging from 7.17% to 9.44%. Furthermore, the role of these nanoparticles as oxygen catalysts has been confirmed to reduce NOx, CO, and unburned hydrocarbon emissions by 7–32% through improved fuel-air mixing.^3^ Beyond their catalytic role, the choice of base fluid itself is consequential, as the hydrocarbon medium governs both the heat-transport behavior and the colloidal persistence of the resulting suspension. n-Dodecane, in particular, is a widely adopted diesel surrogate whose thermophysical and transport properties (including viscosity and thermal conductivity) have been well characterized and validated for combustion modeling,^4,5^ and the dispersion of metal oxide nanoparticles is known to further enhance the effective thermal conductivity of such base fluids.^6^ The base fluid therefore influences nanofuel performance along two coupled axes: its transport properties shape heat transfer, while its temperature-dependent viscosity dictates colloidal stability—the latter being the central focus of this study. However, the translation of these laboratory-scale advantages into practical engineering applications remains constrained by a fundamental obstacle “colloidal instability.”

The stability of a nanofuel is typically characterized by the resistance of dispersed particles to agglomeration and sedimentation over time. In the conventional approach, this issue is addressed through chemical methods such as surfactant addition or pH modification to generate electrostatic or steric repulsion forces between particles. Although chemical stabilization is indispensable for long-term shelf life, recent findings indicate that surfactant efficacy is strictly temperature-dependent.^7,8^ At elevated temperatures, the thermal desorption or degradation of surfactants can compromise these steric or electrostatic barriers, leading to sudden agglomeration. Therefore, rather than focusing on chemical additivation, the present work intentionally isolates transport-driven stability limits to establish the fundamental physical boundaries dictated by the base fluid. In addition, to overcome the limitations of chemical surfactants, there is a growing interest in the literature regarding “dispersant-free” stabilization mechanisms. For instance, a recent study by Lin et al.^9^ elegantly demonstrated that flow-induced dynamic dispersion and particle-mixing strategies can effectively suppress agglomeration and enhance dispersion without relying on any chemical dispersants. Such findings underscore the critical role of physical, structural, and hydrodynamic factors in maintaining colloidal stability, further motivating the present focus on thermophysical and transport-physics-based boundaries.

In high-temperature applications such as organic Rankine cycles (ORC) or fuel injection systems, the thermophysical properties of the base fluid—particularly its viscosity—undergo significant temperature-dependent variation. Minakov et al.^10^ emphasized that these temperature-induced rheological changes directly alter the hydrodynamic forces acting on suspended particles. As noted by Ponticorvo et al.,^11^ failure to manage these variations leads to rapid particle aggregation and a subsequent loss of thermal properties. This variation directly alters the hydrodynamic forces acting on the suspended particles. Maintaining colloidal integrity under these severe thermal gradients is the ultimate prerequisite for achieving the targeted combustion efficiencies in real-world applications.

Despite the extensive literature on surfactant optimization, there is a notable lack of predictive frameworks defining kinetic stability limits as a function of operating temperature and particle size that are experimentally validated under thermal stress. Although particle size (particularly in the 10–50 nm range) and temperature are known to be critical factors,^12^ most existing stability studies are experimental and rely on shelf-life observations at standard ambient temperatures. These traditional static approaches are insufficient to capture the dynamic behavior of the dispersion under thermal stress. Alktranee et al.^13^ have argued that a unified model capable of accurately predicting stability parameters under thermal stress is still lacking. As temperature increases, the strong nonlinear reduction in base fluid viscosity weakens the drag force opposing gravity, potentially rendering a chemically stable suspension kinetically unstable. This mechanism highlights the importance of Brownian motion in resisting sedimentation, which is highly sensitive to the interplay between particle size and viscosity reduction.^14^ Therefore, understanding the mechanistic competition between gravity-induced advective settling and Brownian motion-induced diffusive transport constitutes a fundamental prerequisite for stable nanofuel design.

This study aims to bridge this gap by establishing an experimentally validated thermophysical kinetic stability framework for metal oxide nanofuels in hydrocarbon media. In contrast to the prevailing body of work, which predominantly relies on chemical stabilization and static shelf-life observations recorded at ambient temperature, the present study deliberately isolates the fundamental transport mechanisms that govern stability and resolves the theoretical upper limits imposed by the base fluid itself. To this end, a hybrid methodology is adopted. The first component is an analytical model based on a Lagrangian parametric sweep spanning ten different metal oxide nanoparticles across the 20–100 °C range, from which dimensionless “stability phase maps” are constructed using the Peclet number (Pe). The second component is an experimental program built upon a full-factorial matrix of DLS measurements for three representative oxides (CeO_2_, Fe_2_O_3_, SiO_2_) dispersed in surrogate fuels (iso-octane and n-dodecane), designed to confirm the viscosity-induced instability boundaries predicted by the model.

The resulting framework advances the current understanding of nanofuel stability in three distinct respects. First, it quantitatively identifies and experimentally confirms the temperature-dependent viscosity collapse as the dominant driver of instability, demonstrating how this collapse suppresses the diffusive gain that would otherwise be expected from intensified Brownian motion at elevated temperatures. Second, it translates these transport-physics relationships into a predictive “stability phase map” that functions as a rational design tool, directly yielding the maximum stable particle diameter for a given operating temperature and fuel. Third, it defines and validates a first-order physical stability boundary that establishes a first-order thermophysical ceiling of stability, providing researchers with a transport-based reference limit to consult before resorting to experimentally costly and time-consuming chemical stabilization strategies. Collectively, these contributions reframe nanofuel stability assessment from an empirical, additive-centered exercise into a predictive methodology grounded in transport physics.

## 2 Theoretical Framework and Methodology

In this study, the stability of nanofuel dispersions is modeled by using a Lagrangian approach that examines the behavior of the discrete phase (nanoparticle) within the continuous phase (base fluid) in the dilute single-particle regime. Recent studies have validated the accuracy of this Lagrangian discrete phase modeling approach; for instance, Ambreen et al.^15^ demonstrated that Lagrangian-Eulerian approximations provide high predictive accuracy with maximum deviations as low as 5.34%, while Ramezanpour et al.^16^ successfully utilized this method to integrate multiple force interactions, including Brownian motion. The primary objective of the model is to analyze the equilibrium between macroscopic gravitational forces acting on the particle and microscopic stochastic thermal forces, through the lens of temperature-dependent thermophysical properties.

### 2.1 Model Assumptions and Validity Envelope

To isolate thermophysical transport mechanisms and clarify the physical boundaries of the model, the following validity envelopes have been defined:

- **Ideal Spherical Geometry:** All nanoparticles are explicitly assumed to possess an ideal spherical geometry, which eliminates the additional deviations in the drag coefficient that would otherwise arise from a nonunity sphericity factor. This assumption is not merely a simplification but a deliberate choice that yields a conservative stability estimate: although recent drag formulations confirm that shape parameters critically influence settling behavior,^17^ any departure from sphericity generally increases drag resistance and thereby reduces the settling velocity.^18^ A perfectly spherical particle consequently represents the most mobile, fastest settling case for a given mass, so the resulting boundaries define a lower-bound (worst-case) limit of kinetic stability. Real, nonspherical particles would experience greater hydrodynamic retention and would therefore remain stable at least as long as the present model predicts.
- **Newtonian Fluid Behavior:** Iso-octane and n-dodecane, selected as base fluids, are assumed to exhibit Newtonian behavior within the studied temperature range (293.15–373.15 K). This assumption is grounded in extensive rheological reviews,^19,20^ which confirm that metal oxide nanofluids typically display Newtonian characteristics at low volume fractions (<1%) and transition to non-Newtonian shear-thinning behavior only at higher concentrations.^21^ Rheological deviations potentially observable at high particle loadings are thus neglected under the assumption of a dilute regime.
- **Laminar Flow Regime:** The parameter range is selected such that the particle Reynolds number (*Re_p_* ≪ 0.1) remains within the limits of the Stokes regime. DiBenedetto et al.^22^ explicitly validated the linear drag regime in this low Reynolds number limit for settling particles; thus, inertial forces are assumed to be negligible compared to viscous forces.
- **Chemically Suppressed Agglomeration (Single-Particle Idealization)**: The model inherently assumes a perfectly dispersed, noninteracting state where van der Waals attractive forces and electrostatic repulsions are balanced or chemically suppressed. By neglecting complex particle–particle interactions and aggregation kinetics, the model strictly simulates single-particle transport. Consequently, the proposed phase maps represent an idealized physical baseline (best-case transport scenario). In practical systems where active agglomeration occurs, the effective particle size will increase, which would dynamically shift the predicted stability boundaries and accelerate sedimentation.

### 2.2 Governing Equations

#### 2.2.1 Advective Settling Velocity (Stokes’ Law)

The terminal settling velocity (*V*_*s*_) of a nanoparticle under the influence of gravity is determined by the density difference between the particle and the base fluid, and the viscous resistance of the fluid. While recent molecular dynamics studies suggest that complex interfacial couplings can influence boundary conditions at the nanoscale,^23^ Stokes’ law remains the established fundamental formalism for predicting the terminal velocity of spherical particles in the laminar regime. Accordingly, this velocity is expressed by eq 1:

1 $$V_{s}=\frac{2r_{p}^{2}\left(\rho_{p}-\rho_{f}\left(T\right)\right)g}{9\mu_{f}\left(T\right)}$$

Here, *r_p_* denotes the particle radius, *g* represents the gravitational acceleration, and ***ρ**_p_* indicates the particle density. ***ρ**_f_* (*T*) and ***μ**_f_* (*T*) correspond to the temperature-dependent density and dynamic viscosity of the base fluid, respectively. As evident from the equation, when a temperature rise reduces viscosity (***μ***_*f*_), the settling velocity (*V_s_*) exhibits an increasing trend inversely proportional to the viscosity reduction. This mechanism is consistent with rheological observations in the literature, where the thermal degradation of base fluid viscosity has been confirmed to be a primary driver of accelerated sedimentation.^10,24^

#### 2.2.2 Diffusive Transport (Stokes–Einstein Relation)

The random (Brownian) motion of micro- and nanoscale particles, driven by thermal energy, is characterized by the diffusion coefficient (*D*). Although recent studies suggest that hydrodynamic boundary conditions can influence transport at the nanoscale, the Stokes–Einstein equation remains the fundamental formalism for describing this motion when effective medium viscosity is applied, as given in eq 2:^25^

2 $$D=\frac{k_{B}T}{6{\pi \mu }_{f}\left(T\right)r_{p}}$$

Here, *k*_B_ represents the Boltzmann constant, and *T* denotes the absolute temperature (Kelvin). This equation demonstrates that diffusion is accelerated via a nonlinear coupling effect, driven not only by the temperature rise (*T*) but also significantly by the reduction in viscosity (***μ**_f_*). This synergistic enhancement of particle mobility at elevated temperatures has been numerically corroborated in recent nanofluid dynamics studies.^26^

#### 2.2.3 Stability Index (Peclet Number)

The Peclet number (*Pe*) is employed to nondimensionalize the mechanistic competition between advective transport (sedimentation) and diffusive transport (Brownian motion), characterizing the relative efficiency of microscopic random motion against macroscopic transport scales.^27,28^ In the context of nanofluid stability, *Pe* can be interpreted as the ratio of gravitational potential energy to thermal energy as expressed in eq 3):

3 $$Pe=\frac{V_{s}\cdot \left(2r_{p}\right)}{D}$$

Within this general framework, the condition *Pe* < 1 indicates the “stable/dispersed” regime dominated by Brownian motion, whereas the threshold where *Pe* exceeds unity (Pe > 1) marks the conceptual demarcation point where particle dynamics fundamentally change,^29^ with the system transitioning from equilibrium-like thermal fluctuations to nonequilibrium hydrodynamic interactions,^30^ and ultimately toward gravity-dominated cluster formation.^31^ As elaborated in Section 3.4, this *Pe* = O(1) condition constitutes a universal scaling criterion; the operational stability boundary adopted in this study is consequently expressed through a system-specific critical Peclet contour (*Pe*_crit_) tied to a practical settling tolerance, rather than through an absolute unity crossing.

### 2.3 Decoupling of Electrostatic Interactions

Contrary to prevalent stability studies in the literature, DLVO (Derjaguin–Landau–Verwey–Overbeek) forces are excluded from the model in this study. This constitutes a deliberate methodological preference, rather than a deficiency. Recent literature explicitly distinguishes between colloidal stability, which primarily concerns particle agglomeration,^32^ and kinetic stability, which relates to sedimentation driven by gravitational forces.^33^ While chemical stabilization (via zeta potential >30 mV) effectively hinders interparticle agglomeration, it cannot prevent single-particle sedimentation where gravitational forces dominate hydrodynamic resistance.^34^ Furthermore,^35^ it is emphasized that environmental conditions such as high temperature can compromise these chemical barriers, rendering zeta potential measurements insufficient for predicting dynamic stability under thermal stress.

The objective of this study is to delineate the first-order kinetic boundaries imposed on the particle by the thermophysical properties (viscosity and density) of the base fluid, independent of chemical agents. Consequently, the obtained results represent the physical theoretical ceiling of stability, even under the assumption that the nanofuel is ideally stabilized.

To provide essential context, prior studies on dispersant-assisted nanoparticle stabilization have demonstrated remarkable success in mitigating particle–particle agglomeration. For instance, Kuo et al.^36^ elucidated the high efficiency of specific functional groups (e.g., amino and phosphate groups) in stabilizing TiO_2_ nano powders in organic suspensions, while Chin et al.^37^ demonstrated that tailored diblock copolymer dispersants can provide massive steric repulsion barriers to stabilize nanodiamonds in aqueous systems. While these chemical stabilization mechanisms are profoundly vital for overcoming van der Waals attractions and minimizing cluster formation, they fundamentally address a different aspect of stability. The objective of this present study is to delineate the first-order kinetic boundaries imposed on the particle by the thermophysical properties (viscosity and density) of the base fluid itself. In other words, while chemical dispersants prevent particles from sticking together, the transport-physics-based model of this study defines a first-order physical boundary where the fluid’s reduced viscosity can no longer prevent the gravitational sedimentation of even perfectly dispersed, individual particles. Consequently, the obtained results represent the physical theoretical ceiling of stability, identifying the threshold under thermal stress where reliance on chemical stabilization alone becomes insufficient.

### 2.4 Parametric Analysis Matrix

The developed analytical model was evaluated for combinations of ten different metal oxide nanoparticles and two distinct hydrocarbon fuels across a broad range of temperatures and particle diameters. The general boundaries of the model parameters are summarized in Table 1. To ensure the accuracy and reproducibility of the study, the input parameters were rigorously selected from the literature:

- **Nanoparticle Properties:** The density values of the metal oxide nanoparticles used in the analyses (Table 2) were adopted from standard crystallographic bulk data, a common practice in nanofluid modeling.^38–40^ However, it is acknowledged that slight deviations from bulk density may occur due to lattice parameter expansions at the nanoscale;^41–43^ such secondary effects are neglected in this first-order approximation.
- **Base Fluid Properties:** The temperature-dependent thermophysical variation data of the hydrocarbon base fluids (iso-octane and n-dodecane) (Table 3) were compiled from the literature. Recent molecular dynamics simulations confirm that properties like density and viscosity exhibit substantial variations near the critical point and with temperature rise, necessitating the use of high-fidelity temperature-dependent functions rather than constant values.^44–48^

**Table 1:** Parametric Analysis Variables and Operational Ranges

Parameter	Value/Range
Particle Types	Provided in Table 2.
Base Fluids	Iso-octane (***C***_**8**_ ***H***_**18**_), n-Dodecane (***C***_**12**_ ***H***_**26**_)
Temperature (***T***)	**20–100** °C (Step: **5**)
Particle Diameter (***d*_*p*_**)	**10–100** nm (Step: **2**)
Concentration	Dilute (<**1** vol %)
Total Data Points	**15,640**

**Table 2:** Physical Properties of Metal Oxide Nanoparticles Used in the Model

Nanoparticle	Chemical Formula	Density (g/cm^3^)
Cerium Oxide	CeO_2_	7.13
Copper Oxide	CuO	6.31
Cobalt Oxide	Co_3_O_4_	6.11
Zinc Oxide	ZnO	5.61
Iron Oxide	Fe_2_O_3_	5.24
Manganese Oxide	MnO_2_	5.03
Titanium Dioxide	TiO_2_	4.23
Aluminum Oxide	Al_2_O_3_	3.95
Magnesium Oxide	MgO	3.58
Silicon Dioxide	SiO_2_	2.65

**Table 3:** Temperature-Dependent Thermophysical Properties of Hydrocarbon Base Fluids

	n-Dodecane		Iso-octane	
Temp. (°C)	Density (kg/m^3^)	Viscosity (Pa.s)	Density (kg/m^3^)	Viscosity (Pa.s)
20	748.75	0.00150	695.00	0.00051
30	741.88	0.00125	686.84	0.00045
40	734.93	0.00107	678.52	0.00040
50	727.90	0.00092	670.04	0.00036
60	720.79	0.00080	661.37	0.00032
70	713.58	0.00071	652.52	0.00029
80	706.29	0.00063	643.45	0.00027
90	698.89	0.00057	634.16	0.00024
100	691.38	0.00051	624.62	0.00022

### 2.5 Computational Algorithm and Numerical Solution

The aggregate data set (*N* = 15,640) presented in Supporting Information of this study was derived via a systematic sweep of the solution space, constructed through the Cartesian product of four fundamental parameter sets. The data generation process is predicated on a deterministic parameter space defined by the following parameter sets (*S*) and step sizes (Δ):

- Particle Set (*S_P_*): $\left\{{\mathrm{CeO}}_{2},{\mathrm{Al}}_{2O_{3}},...,{\mathrm{MnO}}_{2}\right\},\left|S_{P}\right|=10$

- Fuel set (*S_F_*): {iso-octane,n-dodecane}, |*S_F_* | = 2.
- Temperature Set (*S_T_*): {*T* ∈ *R*|20 ≤ *T* ≤ 100,**Δ** *T* = 5}, |*S_T_* | = 17
- Diameter Set (*S_d_*): {*d* ∈ *R*|10 ≤ *d* ≤ 100,**Δ** *d* = 2}, |*S*_*d*_| = 46

For each unique state vector *x* = (*p*,*f*,*T*,*d*) ∈ *S_P_* × *S_F_* × *S_T_* × *S_d_* within this defined space, the thermophysical state of the system was computationally resolved utilizing a Python-based deterministic algorithm formulated upon a quasi-steady Lagrangian framework.^15,16^ Given that the characteristic nanoparticle diameters (dp ≥ 10 nm) are sufficiently larger than the molecular diameter of the hydrocarbon solvent, the continuum flow approximation for hydrodynamic drag is justified. This justification merits quantitative substantiation, particularly at the smallest diameter considered (10 nm), where continuum assumptions are most likely to be questioned. Estimating the effective molecular diameter of the base fluids from their molar volume yields values of approximately 0.81 nm for iso-octane and 0.90 nm for n-dodecane, so that even the smallest modeled particle is roughly 11 to 12 times larger than a solvent molecule. If a gas-phase analogy were applied, the corresponding Knudsen numbers would be only Kn ≈ 0.08 at 10 nm and Kn ≈ 0.008 at 100 nm, with nominal Cunningham slip factors of *Cc* ≈ 1.10 and *Cc* ≈ 1.01, respectively. It is essential to recognize, however, that the Cunningham–Knudsen formalism is defined for rarefied gases, in which transport is governed by the molecular mean free path; this construct does not transfer directly to a dense liquid, where molecules are in continuous contact and any interfacial slip arises from atomistic solid–fluid interactions rather than from mean-free-path effects.^49^ In liquid media, deviations from the ideal no-slip Stokes condition are instead governed by the solid–liquid coupling strength and an interfacial density-accumulation length on the order of a few ångström,^23^ and such corrections become appreciable only for particles approaching the molecular scale itself.^50^ For the present size range, the relevant particle-to-molecule size ratio places the system well within the continuum regime. Even under the conservative gas-phase upper bound, the maximum nominal slip correction (∼10%) is confined to the 10 nm extreme and falls below 3% across the 25–40 nm interval where the critical diameters predominantly reside; it therefore does not alter the qualitative form of the predicted boundaries and would, if anything, introduce a slight additional margin of conservatism at the smallest sizes. In this strictly creeping flow regime (*Re_p_* ≪ 0.1), the dynamic response of the discrete phase is entirely dictated by its momentum relaxation time $\left(\tau_{p}=\rho_{p}d_{p}^{2}/18\mu_{f}\right)$ . Because τ*_p_* is bounded on the order of nanoseconds (∼10^‑9^) s), there exists a profound time scale separation between the particle’s instantaneous momentum adjustment and the macroscopic observation periods. Consequently, the transient inertial term (*m_p_d*v⃗/*dt*), along with the Basset history and added-mass forces within the generalized Boussinesq-Basset-Oseen (BBO) equation of motion,^51^ become infinitesimally small. While recent comprehensive reviews emphasize that neglecting the history force requires rigorous case-by-case justification rather than blanket assumptions,^52^ the extreme time scale separation established here physically validates this omission. Integrating these trajectory equations via explicit temporal discretization (**Δ** *t* time-stepping) would not only be physically redundant but would also introduce severe numerical stiffness (stiff ODEs). Adopting the quasi-steady state approximation (QSSA) effectively circumvents this numerical stiffness without altering the terminal physical state of the system.^53^ Therefore, the Lagrangian tracking is mathematically reduced to an instantaneous force equilibrium, assuming the particle perpetually resides at its quasi-steady terminal transport state. By decoupling the stochastic thermal fluctuations (Brownian diffusion) from the deterministic body forces (gravitational advection), the algorithm independently evaluates the superposed transport rates. To systematically map these kinetic stability limits, the code executes a highly discretized, nested multidimensional sweep across the specified state-space matrix. At each distinct iterative step, the computational protocol dynamically couples the temperature-dependent rheological state of the continuous phase to the deterministic hydrodynamic response of an isolated single-particle frame, according to the following sequence of steps:

**Update of Thermophysical Properties:** In the initial step, the density (ρ_f_(*T*)) and viscosity (μ*f*(*T*)) of the base fluid corresponding to the selected operating temperature (*T*) are computed via cubic spline interpolation of the experimental data provided in Table 3. This interpolation method was selected to minimize numerical dissipation and ensure a smooth representation of nonlinear property variations, offering superior accuracy compared to linear methods for thermodynamic data.^54,55^

**Force Balance and Terminal Velocity:** Under the assumption of the Stokes regime, the terminal settling velocity (*V_s_*) is derived via eq 1 by balancing the net gravitational force acting on the particle with the viscous drag force.
**Diffusive Mobility:** The corresponding temperature and viscosity values are utilized in the Stokes–Einstein equation (eq 2) to determine the Brownian diffusion coefficient (*D*) of the nanoparticle.
**Nondimensionalization and Decision:** The instantaneous Peclet number (*Pe*) of the system is derived via eq 3 using the calculated *V_s_* and *D* values. The algorithm compares the *Pe* number and the settling velocity against predetermined stability thresholds (***Pe**_crit_* and ***V**_limit_* = **1** mm/day) to categorize the instantaneous system state into one of the “Stable”, “Transition”, or “Unstable” phases, subsequently recording it into the global data matrix (*M*).

The total number of data points generated through this systematic sweep can be verified by the product of the cardinalities of the parameter sets:

$$N_{total}=\left|S_{P}\right|\times \left|S_{F}\right|\times \left|S_{T}\right|\times \left|S_{d}\right|=10\times 2\times 17\times 46=15,640$$

All of the calculations were performed using double-precision arithmetic. This methodological approach demonstrates that the study is grounded in the deterministic solution of a defined parameter space, rather than random sampling. Recent computational studies highlight that deterministic methods offer superior reproducibility and convergence in defining exact stability boundaries compared to stochastic approaches.^56,57^

### 2.6 Experimental Validation Methodology

To validate the critical stability boundaries and viscosity-induced instability mechanisms predicted by the analytical model, we conducted a comprehensive experimental study. The experimental design was structured to isolate the effects of particle density and base fluid viscosity under thermal stress independent of commercial fuel additives.

#### 2.6.1 Materials

All reagents utilized in the validation experiments were of analytical grade and were used without further purification to ensure thermophysical consistency. n-Dodecane (Product No: 8.20543) and isooctane (Product No: 1.04727) were selected as high-purity surrogate base fluids to represent diesel and gasoline, respectively.

For the discrete phase, three representative metal oxide nanoparticles were selected to cover the low, medium, and high-density regimes defined in the theoretical model:

- **High Density:** Cerium­(IV) oxide (CeO_2_, Product No: 544841), density: ∼7.13 g cm^–3^, particle size <25 nm (BET).
- **Medium Density:** Iron­(III) oxide (Fe_2_O_3_, Product No: 544884), density: ∼5.24 g cm^–3^, particle size <50 nm.
- **Low Density:** Silicon dioxide (SiO_2_, Product No: 637238), density: ∼2.65 g cm^–3^, particle size 10–20 nm (BET), 99.5% trace metals basis.

All materials were purchased from Sigma-Aldrich. The primary criterion for selecting these specific oxides was to provide a broad physical representation of the density parameter space (2.65–7.13 g cm^–3^), thereby enabling the observation of density-driven hydrodynamic deviations predicted by the Phase Maps.

#### 2.6.2 Sample Preparation and Dispersion Protocol

Nanofuel suspensions were prepared at a fixed mass concentration of 100 ppm in a total volume of 100 mL for each experimental condition. The selection of the 100 mL volume was critical to ensure effective vortex formation during mechanical stirring and to prevent localized overheating during ultrasonication, which is a common artifact in smaller sample volumes. The dispersion protocol was conducted in two stages:

**Mechanical Premixing:** Crude suspensions were initially homogenized using a high-speed mechanical stirrer at 15,000 rpm for 10 min to break down macroagglomerates and ensure uniform wetting of the nanoparticle surface.
**Ultrasonic Homogenization:** Following premixing, the samples were subjected to ultrasonication using a probe-type homogenizer (20 kHz, 100 W) for 10 min. To strictly control the temperature and prevent thermally induced reagglomeration during the process, the samples were maintained in an ice bath, ensuring the bulk temperature remained below 10 °C.

#### 2.6.3 Characterization and Measurements

Prior to quantitative analysis, a qualitative dispersion check was performed using optical microscopy to ensure no visible macro-flocculation was present. The hydrodynamic particle size distribution (*Z*-Average) and Polydispersity Index (PDI) were measured using Dynamic Light Scattering (DLS) (Malvern Zetasizer Nano ZS). Zeta potential (**ζ**) measurements were conducted using the same instrument based on the principle of electrophoretic light scattering.

To simulate the thermal operating conditions modeled in the study, the measurement cell temperature was stabilized at three distinct points: 20 °C, 50 °C, and 80 °C. For each measurement, the specific temperature-dependent viscosity and refractive index of the base fluids were manually input into the analyzer to ensure the accuracy of the Stokes–Einstein calculation. Each reported value corresponds to the instrument-averaged result of multiple sequential runs automatically performed by the analyzer for a single freshly prepared specimen. Since the objective of the experimental campaign was to verify the relative density- and temperature-dependent stability trends predicted by the model rather than to establish absolute electrokinetic values, the magnitude of the observed variations in hydrodynamic diameter and potential far exceeds the typical measurement uncertainty, and the reported data are accordingly interpreted in a semiquantitative manner.

#### 2.6.4 Experimental Matrix

A full-factorial experimental matrix consisting of 18 unique conditions was executed, comprising:

- **2 Base Fluids:** Iso-octane (low viscosity/gasoline surrogate) vs n-dodecane (high viscosity/diesel surrogate).
- **3 Nanoparticles:** CeO_2_, Fe_2_O_3_, and SiO_2_ (varying densities).
- **3 Temperatures:** 20 °C (Ambient), 50 °C (Transition), 80 °C (High thermal stress).

This matrix approach allows for a direct systematic comparison between the theoretical predictions and the actual physical behavior of the nanofuels under thermal stress.

## 3 Results and Discussion

In this section, the kinetic stability behavior of metal oxide nanoparticles in a hydrocarbon environment is analyzed via a developed thermophysical model. The discussion is structured not only around observed trends but also on the characteristic time scales, sensitivity gradients, and analytical scaling laws governing these trends.

### 3.1 Characteristic Time Scales and Thermophysical Coupling

The temperature-dependent evolution of nanofuel stability is fundamentally a consequence of the proportional shift between the diffusive propagation time scale (***τ**_diff_*) and the advective settling time scale (***τ***_*sed*_). Experimental studies confirm that these sedimentation dynamics are governed by multiple metastable states, where temperature-induced viscosity changes can dramatically alter the suspension behavior.^58^ Simulation data indicate that an increase in temperature from 20 to 100 °C induces a significant reduction in iso-octane viscosity within the range of approximately ∼55–65%. This observation aligns with the findings of Kulkarni et al.^59^ who demonstrated that nanofluid viscosity decreases exponentially with increasing temperature. This rheological alteration affects the system dynamics in two opposing directions, as illustrated in Figure 1:

- **Diffusive Gain:** The increase in thermal energy (*k_B_T*) shortens the diffusion time $\left(\tau_{diff}∼L_{c}^{2}/D\right)$ required for the particle to traverse a characteristic distance. The blue curve in Figure 1 (diffusion time) substantiates that particles become thermally more mobile with rising temperature.Loulijat and Moustabchir^60^ attribute this phenomenon to the intensification of Brownian motion, which acts as a mechanism to enhance particle mobility.
- **Hydrodynamic Collapse:** The reduction in viscosity weakens the Stokes drag force, drastically shortening the settling time (τ*_sed_* ∼ *L_c_*/*V_s_*). This “hydrodynamic collapse,” represented by the red curve in Figure 1, signifies the attenuation of the fluid’s capacity to maintain particle suspension. A similar instability trend was observed by^61^ for n-dodecane systems, where elevated temperatures were found to accelerate droplet growth and instability mechanisms due to reduced continuous phase viscosity.

**Figure 1:**
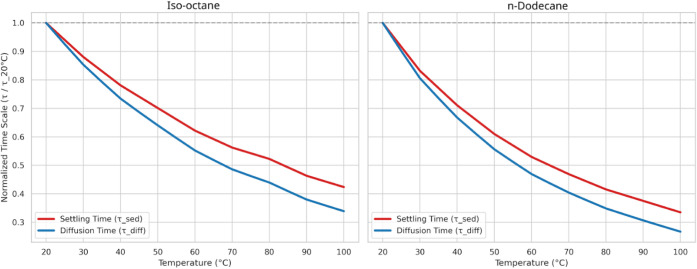
Temperature-dependent evolution of normalized time scales (*τ*/*τ*_20 °_ *C*) for iso-octane and n-dodecane-based nanofuels. Blue curves represent the increase in diffusive mobility (decrease in diffusion time), while red curves represent the loss in hydrodynamic retention capacity (decrease in settling time). The steeper slope of the red curve indicates that the dominant mechanism of instability is viscosity degradation.

The analyses reveal that as temperature increases, both time scales decrease rapidly compared to their reference values at 20 °C. This “race of time scales”, observable in Figure 1, demonstrates that although the system is thermodynamically more mobile at elevated temperatures, it becomes temporally more vulnerable to advective transport in the direction of the gravity vector. While the temperature-dependent reduction of fluid viscosity is a well-established rheological phenomenon, the novelty of the present framework lies in quantifying how this specific reduction dictates the transition between Brownian-dominated and gravity-dominated regimes. By mapping these well-known thermophysical trends onto a dimensionless Peclet space, the study transforms qualitative rheological knowledge into a predictive tool that identifies the precise coordinates where hydrodynamic support fails at the nanoscale. This aligns with Minakov et al.^10^ who concluded that while Brownian motion is significant, the overall rheological and stability properties are critically determined by the viscosity reduction at high temperatures. These comparative data obtained for iso-octane and n-dodecane analytically corroborate that stability is determined not solely by energy balance but by the competition of transport rates driven by the hydrodynamic weakening caused by viscosity degradation.

### 3.2 Phase Maps and Sensitivity Gradients

The combined effect of particle density and operating temperature can be discerned from the gradients of the isolines (iso-value contours) within the phase maps presented in Figure 2. These maps demonstrate that the mathematical character of the system’s response to thermal stress undergoes a fundamental shift as a function of the particle density difference (**Δ** *ρ*). This distinction is critical, as recent comparative studies have confirmed that density variations can induce up to a 400% change in terminal settling velocity, far exceeding the partial effects of temperature alone.^62^

- **Low-Density Regime (SiO**_**2**_ **):** The stability contours (green regions) in the phase map exhibit a behavior characterized by a flat gradient, running nearly parallel to the temperature axis. This indicates that the small magnitude of the **Δ** *ρ* term acts as a dampening effect on viscosity variations (**μ**(*T*)), implying that the system exhibits low sensitivity to temperature fluctuations. This aligns with the findings of Oshima et al.^63^ who demonstrated that lighter particles like SiO_2_ display distinctly stable settling characteristics compared to denser metal oxides.
- **High-Density Regime (CeO**_**2**_ **):** Conversely, for heavier particles, the stability boundaries display a steep gradient. Mathematically, this is attributed to the fact that the derivative sensitivity of the Stokes velocity to viscosity changes (∂*V_s_*/∂) scales directly with the density difference (**Δ** *ρ*). Mu et al.^64^ observed similar complex instability dynamics for CeO_2_ dispersions, noting their high susceptibility to aggregation and settling. While density acts as a linear first-order multiplier increasing sensitivity, the exponential decay of viscosity remains the dominant temperature-coupled variable driving the instability. Consequently, the sharp transition observed above 60 °C for CeO_2_ in iso-octane represents not merely an acceleration in velocity, but a regime shift where the gravitational potential becomes directly dominant following the collapse of the system’s viscosity barrier.

**Figure 2:**
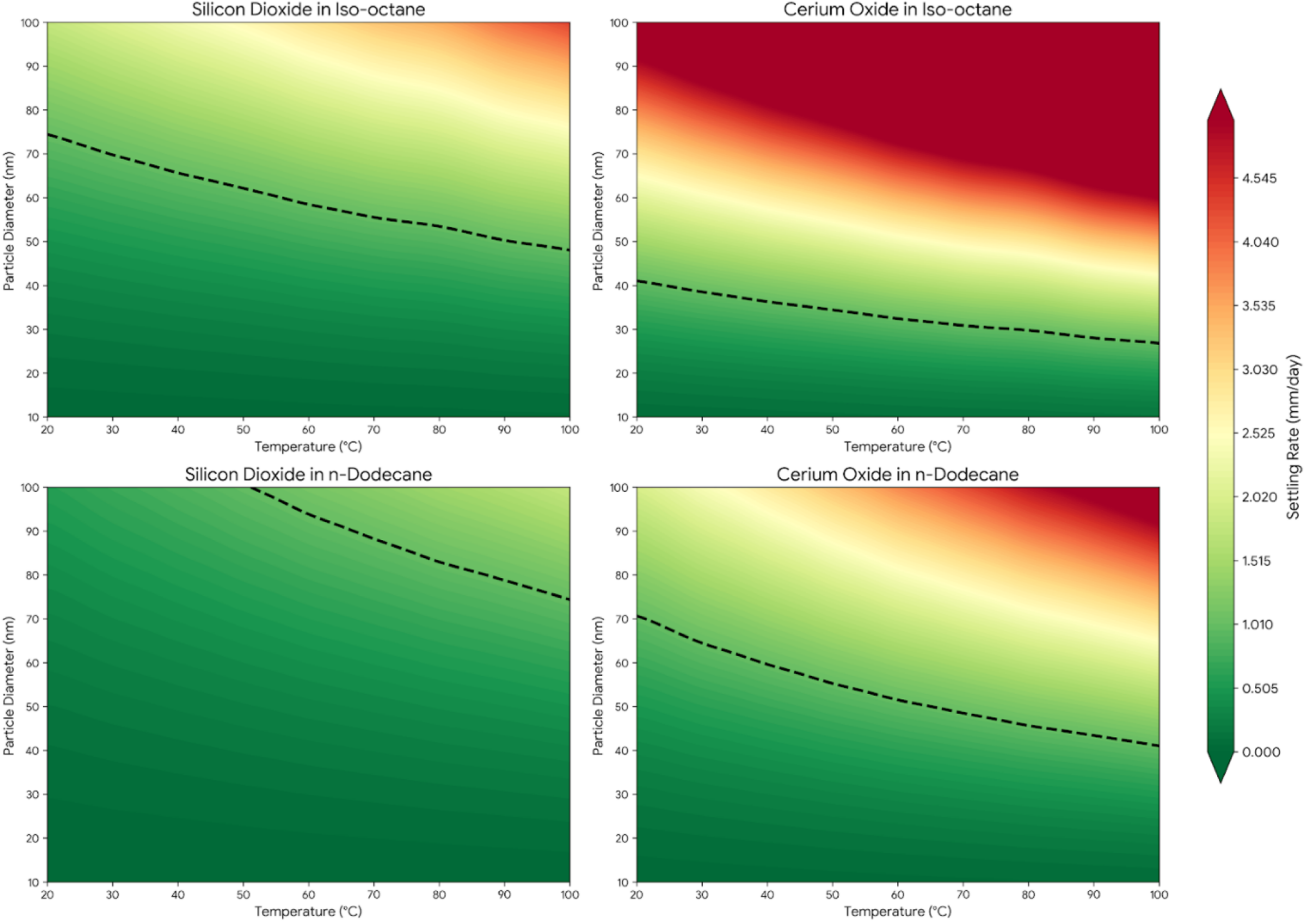
Kinetic stability phase maps generated for cerium oxide (CeO_2_, high density) and silicon dioxide (SiO_2_, low density) nanoparticles. The black dashed line demarcates the critical phase boundary separating the safe operating zone (stable) from the risk zone (unstable).

### 3.3 Analytical Behavior of Critical Boundary Curves

The Critical Stability Limits derived for engineering design purposes (Figure 3) represent an analytical projection of Stokes’ law, rather than a mere empirical observation. As extensively documented in the literature, universally agreed-upon maximum settling velocity thresholds are rarely specified, as stability limits are highly formulation-dependent. Instead, the long-term kinetic stability of commercial slurries and nanofuels is conventionally characterized by monitoring macroscopic phase separation and settling distance over periods ranging from days to months.^58,65^ To bridge the gap between these observational shelf-life practices and analytical modeling, a rigorous practical tolerance of *V*_*s*_ ≤ 1 mm/day was adopted in this study. This specific criterion is not arbitrary; it translates to a macroscopic sedimentation of approximately 3 cm over a standard one-month storage cycle. This magnitude serves as an acceptable conservative baseline for kinetic shelf-life stability, ensuring that the bulk nanofuel maintains adequate macroscopic homogeneity without requiring constant mechanical redispersion during typical logistics. For this practical engineering threshold, the relationship between the critical particle radius (*r_crit_*) and viscosity can be derived as follows (eq 4):

4 $$r_{crit}=\sqrt{\frac{9\mu_{f}\left(T\right)V_{limit}}{2\left(\rho_{p}-\rho_{f}\left(T\right)\right)g}}$$

**Figure 3:**
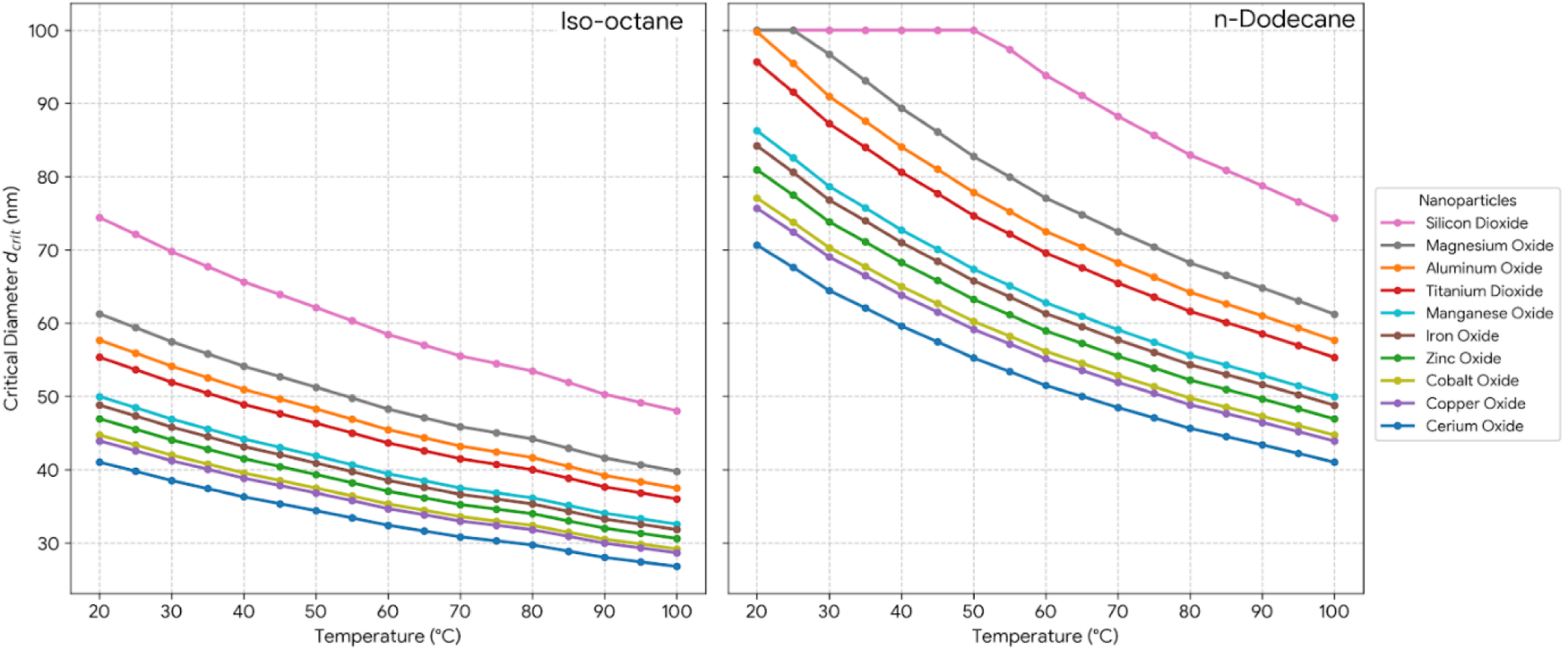
Critical boundary curves indicating the temperature-dependent maximum stable particle diameter (*d_crit_*) for hydrocarbon-based nanofuels. The curves are derived based on a 1 mm/day settling velocity tolerance and validate that the theoretical stability ceiling decays in correlation with √μ. The markers denote values of the closed-form analytical expression (eq 4) evaluated at discrete 5 °C intervals, while the connecting lines represent the underlying continuous function rather than an interpolation of experimental data points.

This relation elucidates why the curves in Figure 3 exhibit a square-root characteristic decay rather than a linear trend.

- **Thermal Evolution:** For iso-octane-based CeO_2_, the decrease in critical diameter from ∼41 nm at 20 °C to ∼27 nm at 90 °C is directly proportional to the square root of the base fluid’s viscosity curve $\left(\sqrt{\mu }\right)$ . This analytical finding confirms that the curve is not arbitrary; rather, it physically represents the thermal evolution of the fluid’s momentum transport capacity.
- **System-Specific Theoretical Baseline:** Consequently, the ∼35 nm boundary identified in this study should not be interpreted as a universal physical threshold but rather as a system-specific idealized estimate for the “effective hydrodynamic support” capacity in these specific low-viscosity hydrocarbon media. In practical colloidal systems, this theoretical boundary will inevitably be influenced by particle–particle interactions, aggregation kinetics, and surface chemistry, which can shift the effective stability limits. Nevertheless, this system-specific baseline aligns well with the “critical size phenomenon” reported by Chen et al.^66^ who observed that nanoparticles smaller than 50 nm are fundamentally required for effective stabilization in low-viscosity fluids. Furthermore,Mukherjee et al.^14^ confirmed that reducing particle diameter below this range is essential for Brownian motion to effectively counteract sedimentation. Beyond this idealized limit, sole reliance on chemical stabilization (surfactants) is unlikely to ensure stability due to the overwhelming magnitude of physical forces. Because the critical diameter is anchored to the adopted 1 mm/day settling tolerance, the sensitivity of the predicted boundaries to this engineering criterion warrants explicit examination. The functional form of eq 4 dictates that the critical radius scales with the square root of the velocity tolerance (r_crit_ ∝ √V_limit_), so the boundaries respond to changes in the criterion in a damped, sublinear manner rather than diverging sharply. This behavior is quantified in Table 4 for the representative CeO_2_/iso-octane system across three plausible tolerances spanning a 4-fold range (0.5, 1.0, and 2.0 mm/day). A 4-fold variation in the velocity criterion translates into only a 2-fold change in the critical diameter (a factor of √4), and although the absolute values shift, the qualitative ordering and the temperature-dependent contraction of the stable window are preserved across all three criteria. The 1 mm/day value therefore functions as a representative midpoint whose specific choice does not materially alter the conclusions of the study; a stricter or more permissive tolerance would shift the boundaries predictably without changing the governing density- and temperature-dependent trends.

**Table 4:** Sensitivity of the Predicted Critical Diameter (Dcrit) to the Adopted Settling-Velocity Tolerance, Shown for the Representative CeO_2_/Iso-octane System^a^

Temp. (°C)	d_crit_ at 0.5 mm/day (nm)	d_crit_ at 1.0 mm/day (nm)	d_crit_ at 2.0 mm/day (nm)
20	29.0	41.0	58.0
50	24.3	34.4	48.6
80	21.0	29.7	42.0

a The critical diameter scales as √*V*_*limit*_, so a fourfold change in the tolerance (0.5–2.0 mm/day) produces only a twofold change in *d*_*crit*_, while the temperature dependent trend is preserved across all criteria.

### 3.4 Peclet Number and Theoretical Framework

Finally, the Peclet number (*Pe*) establishes the dimensionless theoretical framework for the time scales and critical limits discussed in previous sections. Analyses demonstrate that the empirically defined “1 mm/day” threshold corresponds to a specific critical Peclet contour (*Pe_crit_*) in dimensionless space. To explicitly decode the underlying physics, Figure 4 maps the entire computed state-space (N = 15,640) onto a logarithmic coordinate system, where the *x*-axis represents the dimensionless Peclet number and the *y*-axis denotes the macroscopic settling rate (*V_s_*).

**Figure 4:**
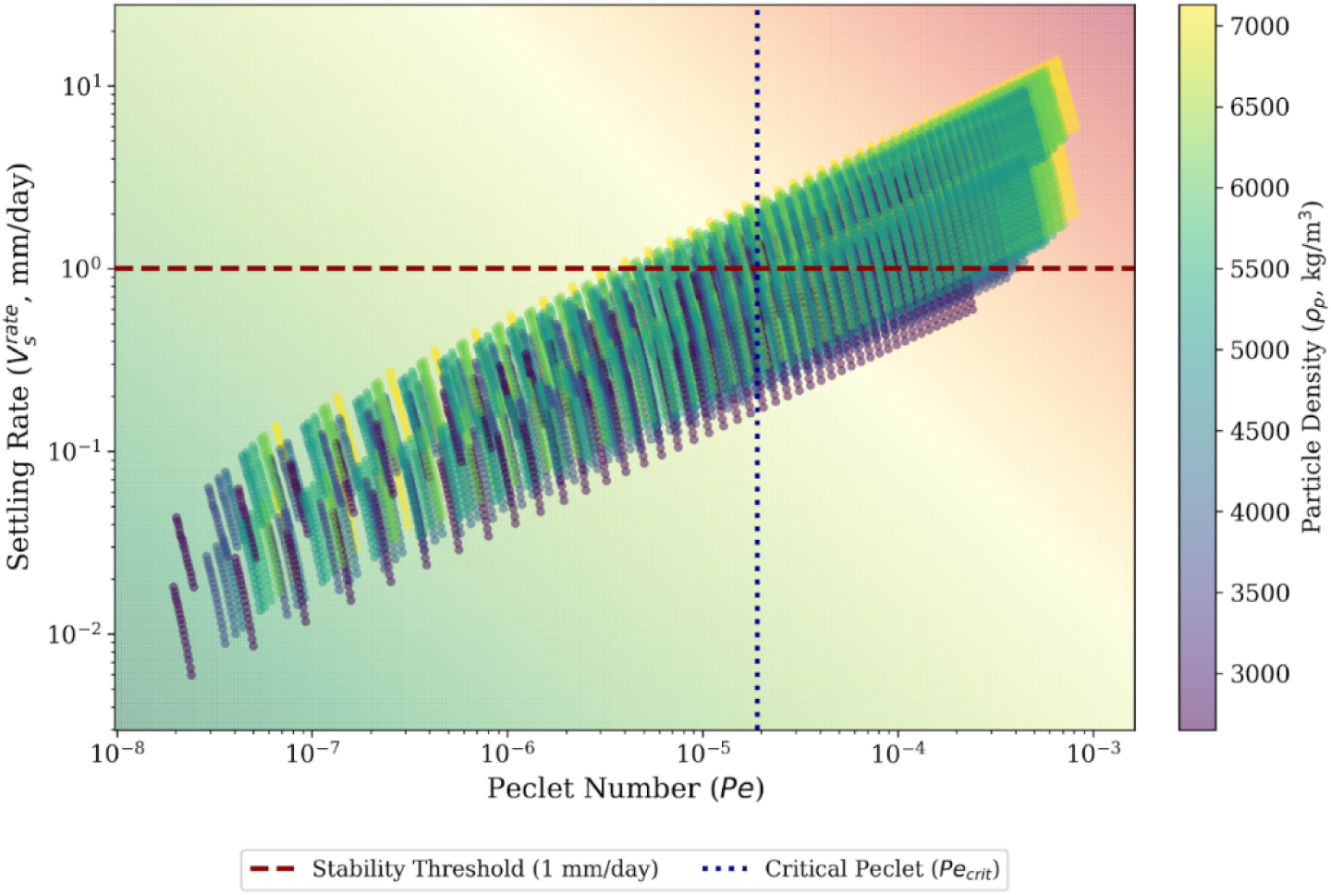
Peclet regime analysis and stability decision map. The log–log plot demonstrates the correlation between the dimensionless Peclet number (*Pe*) and the macroscopic settling rate. The colormap explicitly decodes the role of particle density (**ρ**_*p*_). Denser particles (yellow markers, e.g., CeO_2_) are stratified at the critical upper boundary of the data band, making them highly susceptible to crossing the orthogonal instability thresholds (dashed and dotted lines) under thermal viscosity collapse. Lighter particles (purple markers, e.g., SiO_2_) maintain a robust safety margin within the Brownian-dominated stable regime (green background).

The linear correlation observed within this log–log space confirms that as the system’s thermal ratio (*Pe*) increases, the macroscopic advective failure accelerates proportionally. More importantly, the color gradient in Figure 4 isolates the specific impact of particle density (**ρ** *_p_*), revealing a strict internal stratification within the data band. Particle density acts as a severe mathematical multiplier in this transport balance. Because the settling velocity is directly proportional to the density difference (*V_s_* ∝ **Δ**), and the Peclet number itself is a function of this velocity (*Pe* = *V_s_d_p_*/*D*), heavier particles suffer a “double physical penalty.” This is visually captured by the yellow/lighter markers (representing high-density oxides like CeO_2_, ∼7130 kg/m^3^), which consistently populate the uppermost frontier of the point cloud.

When elevated thermal stress induces base fluid viscosity collapse, these high-density states rapidly traverse from the Brownian-dominated “Stable Zone” (green background) and breach the orthogonal intersection of *V_s_* =1 mm/day and *Pe* = *Pe_crit_* into the “Unstable Zone” (red background). Conversely, the dark purple markers (representing low-density oxides like SiO_2_, ∼2650 kg/m^3^) remain deeply anchored at the bottom-left of the distribution, retaining a massive margin of safety against thermal degradation. In the conceptual framework of colloidal transport, the shift from a diffusion-dominated to a gravity-dominated regime is associated with the Peclet number approaching order unity; Poydenot et al.^67^ characterize the *Pe* ≪ 1 domain as a “Brownian-dominated” regime governed by random thermal motion, whereas Yun et al.^68^ identify *Pe* ≫ 1 as the regime where gravitational sedimentation dictates particle dynamics. It should be stressed, however, that this *Pe* = O(1) criterion represents a universal scaling boundary rather than the operational limit of the present systems: because the absolute Peclet numbers computed across the entire state-space remain well below unity, the practically relevant transition is governed by the system-specific critical contour (*Pe_crit_*) defined below, at which advective settling overtakes diffusion under the adopted engineering tolerance.

The “safe zones” depicted in the phase maps essentially represent regimes where diffusion dominates the settling motion. Phung et al.^69^ and Dhas and Roy^70^ have theoretically confirmed that maintaining the system within a low-Peclet envelope significantly delays instability, while an approach toward order-unity Peclet values marks the conceptual onset of hydrodynamic destabilization. Within the specific low-Peclet envelope of the present hydrocarbon systems, this universal principle manifests as the system-specific *Pe_crit_* contour rather than as an absolute *Pe* = 1 crossing. The individual transport relations underlying this analysis—Stokes settling, Stokes–Einstein diffusion, and their ratio through the Peclet number—are themselves well established, and the contribution of the present framework lies not in these constituent relations but in the way they are combined, parametrized, and applied. Earlier dimensionless treatments of suspension stability have typically been framed around convective or porous-media descriptors, such as the Darcy–Rayleigh number, and have generally aimed at characterizing the onset of bulk convective motion rather than the kinetic fate of individual particles.^71,72^ In contrast, the framework developed here takes the temperature-dependent viscosity collapse of the base fluid as its explicit organizing axis and recasts the Peclet criterion into a two-dimensional decision surface that returns, for any operating temperature and oxide density, the maximum particle diameter compatible with kinetic stability. This reorientation converts a familiar dimensionless ratio into a direct design tool rather than a descriptive index, and—unlike most prior dimensionless stability analyses—the resulting boundaries are corroborated experimentally under thermal stress. The limits presented in this study therefore constitute not merely engineering tolerances, but generalizable regime transition boundaries that are both grounded in fundamental transport physics and validated against measured behavior.

It is important to clarify the status of the critical Peclet contour (*Pe_crit_*) employed here, as the resulting phase boundaries depend directly upon it. Rather than being an externally calibrated or fitted parameter, *Pe_crit_* is determined internally and deterministically: it is the locus of states in the dimensionless Peclet–settling-rate space that coincides with the practical 1 mm/day settling tolerance adopted in Section 3.3. Because both *V*_*s*_ and the Peclet number are computed from the same closed-form transport relations, this contour emerges directly from the governing physics rather than from any empirical adjustment. The experimental observations are consistent with this construction: the high-density CeO_2_/iso-octane system, whose computed states lie closest to the *Pe_crit_* contour at elevated temperature, is precisely the system observed to destabilize first (at 50 °C), whereas the low-density SiO_2_ system, situated far below the contour across the entire temperature range, remains experimentally stable throughout. This correspondence indicates that the contour is not an arbitrary cutoff but a physically meaningful boundary whose predictions track the observed onset of instability. The robustness of the resulting boundaries to the underlying tolerance is examined explicitly in the sensitivity analysis presented below.

### 3.5 Experimental Validation of Theoretical Limits

To corroborate the analytical boundaries defined by the Stability Phase Maps, the hydrodynamic diameter (*D_h_*), Polydispersity Index (PDI), and Zeta potential (**ζ**) of the selected metal oxide nanofuels were measured under thermal stress conditions. The experimental results, summarized in Table 5, provide concrete evidence for the viscosity-induced instability mechanism proposed in this study. It is important to emphasize that hydrodynamic diameter and Zeta potential are utilized here not as parameters within the modeling framework, but as independent diagnostic indicators to verify the theoretical predictions. Since the model defines an idealized first-order ceiling for stability, the experimental observation of Zeta potential collapse and runaway agglomeration serves to demonstrate the real-world physicochemical consequences that occur once this hydrodynamic limit is breached. It is important to note that DLS measures the hydrodynamic diameter (*D_h_*), which includes the solvation shell and soft agglomerates. Therefore, the measured values are inherently larger than the primary crystallite sizes used in the analytical model (<**25** nm). However, the relative growth trends provide a direct validation of the stability physics.

**Table 5:** Experimental Validation Results for Metal Oxide Nanofuels under Thermal Stress^a^

Test	Base Fluid	Particle	Temp. (°C)	Viscosity (Pa.s)	Dh (nm)	PDI	Ζeta P(mV)	Status
T01	Iso-octane	CeO_2_	20	0.00051	85.8	0.153	–26.8	Stable
T02	Iso-octane	CeO_2_	50	0.00036	239.5	0.355	–18.4	Unstable
T03	Iso-octane	CeO_2_	80	0.00027	621.6	0.627	–8.7	Unstable
T04	Iso-octane	Fe_2_O_3_	20	0.00051	125.8	0.171	–24.1	Stable
T05	Iso-octane	Fe_2_O_3_	50	0.00036	219.8	0.251	–19.6	Metastable
T06	Iso-octane	Fe_2_O_3_	80	0.00027	400.8	0.447	–13.9	Unstable
T07	Iso-octane	SiO_2_	20	0.00051	44.8	0.117	–31.7	Stable
T08	Iso-octane	SiO_2_	50	0.00036	45.0	0.130	–30.4	Stable
T09	Iso-octane	SiO_2_	80	0.00027	47.3	0.136	–28.9	Stable
T10	n-Dodecane	CeO_2_	20	0.00150	82.4	0.144	–29.6	Stable
T11	n-Dodecane	CeO_2_	50	0.00092	89.5	0.180	–27.3	Stable
T12	n-Dodecane	CeO_2_	80	0.00063	123.8	0.258	–21.4	Metastable
T13	n-Dodecane	Fe_2_O_3_	20	0.00150	116.9	0.166	–25.8	Stable
T14	n-Dodecane	Fe_2_O_3_	50	0.00092	127.7	0.173	–24.2	Stable
T15	n-Dodecane	Fe_2_O_3_	80	0.00063	143.4	0.221	–19.1	Stable
T16	n-Dodecane	SiO_2_	20	0.00150	43.6	0.108	–34.6	Stable
T17	n-Dodecane	SiO_2_	50	0.00092	43.9	0.109	–33.9	Stable
T18	n-Dodecane	SiO_2_	80	0.00063	45.5	0.119	–32.5	Stable

a (*D_h_* represents the *Z*-average hydrodynamic diameter, and PDI denotes the polydispersity index).

A conceptual clarification is warranted regarding the correspondence between the single-particle transport model and aggregation-based experimental observables. The analytical framework does not attempt to simulate the growth kinetics of clusters; rather, it predicts the thermophysical threshold at which an isolated perfectly dispersed particle can no longer be hydrodynamically supported by the base fluid. Aggregation and the subsequent macroscopic settling are not treated here as the processes being modeled but as the physical consequences that are expected once this threshold is crossed. As the temperature-induced viscosity collapse weakens the Stokes drag, the hydrodynamic resistance that keeps individual particles separated diminishes, allowing attractive interactions to dominate and trigger the runaway growth captured by DLS. Within this logic, the measured increase in hydrodynamic diameter does not characterize a process distinct from the one modeled; it constitutes the observable downstream signature of the single-particle stability ceiling being breached. The experiments therefore validate the model not by reproducing its single-particle idealization but by confirming that the onset of instability coincides with the predicted thermophysical boundary.

#### 3.5.1 Impact of Base Fluid Viscosity (The Surrogate Effect)

The experimental data reveal a stark contrast in stability behavior between the gasoline surrogate (iso-octane) and the diesel surrogate (n-dodecane), driven primarily by their viscosity differentials.

- **Iso-octane Case (Low Viscosity):** As predicted by the model, CeO_2_ nanoparticles in iso-octane (Test T03) exhibited a catastrophic stability failure at 80 °C. The hydrodynamic diameter surged from 85.8 nm at 20 °C to 621.6 nm at 80 °C, accompanied by a sharp rise in PDI to 0.627. This confirms that the severe reduction in iso-octane viscosity (**μ** ≈ 0.27cP at 80 °C) strips away the hydrodynamic drag barrier, leading to rapid collision and agglomeration.
- **n-Dodecane Case (High Viscosity):** In contrast, the same CeO_2_ nanoparticles in n-dodecane (Test T12) maintained a significantly smaller diameter (123.8 nm) under the same thermal load. Since n-dodecane retains a higher viscosity (**μ**≈ 0.63cP) even at 80 °C, it successfully dampens the Brownian collisions, preventing the runaway agglomeration observed in the lighter fuel.

#### 3.5.2 Density-Driven Sensitivity

The results validate the “Density Sensitivity” hypothesis depicted in Figure 2.

- **High-Density (CeO**_**2**_, **7.13 g cm** ^**–3**^ **):** Exhibited the highest sensitivity to thermal stress, crossing the critical stability threshold in iso-octane at 50 °C (Test T02).
- **Low-Density (SiO**_**2**_, **2.65 g cm** ^**–3**^ **):** Demonstrated remarkable immunity to temperature-induced viscosity loss. As shown in Tests T07–T09, the particle size of SiO_2_ remained virtually constant (∼45–47 nm) even when the temperature was raised to 80 °C. This empirical finding aligns with the “flat gradient” stable zones predicted for lighter oxides in the theoretical phase maps. The pronounced influence of particle size on the colloidal persistence of silica suspensions has likewise been documented in aqueous systems, where the temporal evolution of SiO_2_–H_2_O nanofluid stability and wettability depends strongly on the primary particle size,^73^ and where the stability of silica nanofluids has been characterized in conjunction with their wetting behavior.^74^ Although those studies concern aqueous media and interfacial wettability rather than the transport-driven sedimentation examined here, the convergence is instructive: in both contexts, the favorable stability of silica is governed primarily by its small effective size and low density, which together keep the diffusive contribution dominant. The present results extend this size- and density-controlled behavior into low-viscosity hydrocarbon media and frame it within a temperature-dependent transport-physics boundary.

While the macroscopic settling behavior strongly aligns with the density-driven sensitivity predicted by the phase maps, it is imperative to acknowledge that these three metal oxides (CeO_2_, Fe_2_O_3_, and SiO_2_) also possess substantially different physicochemical properties. As extensively reviewed in the literature, metal oxides exhibit distinct isoelectric points (IEPs) and surface hydroxyl densities,^75,76^ which critically dictate their initial electrokinetic state. These surface chemistry variations directly govern the structure of the electrical double layer (EDL) and subsequent aggregation kinetics, since maximum agglomeration typically occurs near the IEP, where electrostatic repulsion is neutralized.^77,78^ Furthermore, specific fluid-particle interactions and the formation of a solvation shell strongly influence the effective hydrodynamic diameter (*D*_h_) measured via DLS, causing inherent deviations from the dry core size.^79,80^ Therefore, while particle density establishes the first-order macroscopic gravitational limit (the settling ceiling), the microscopic aggregation dynamics driving the particles toward this ceiling are inherently modulated by these complex surface properties. The observed experimental deviations from an ideal, purely physical model underscore the reality that density acts as an amplifier of instability, but the onset of aggregation is a coupled thermophysical-chemical process.

This experimental design also allows the hydrodynamic contribution to be distinguished from the electrokinetic one, even though both vary with temperature. A reduction in zeta potential magnitude with increasing temperature is observed for all three oxides, including SiO_2_, whose potential decreases from −31.7 to −28.9 mV between 20 and 80 °C in iso-octane. If a temperature-driven weakening of electrostatic stabilization were the controlling mechanism, SiO_2_ would be expected to destabilize accordingly; instead, it retains a nearly constant hydrodynamic diameter (∼45 nm) across the entire range, whereas the high-density CeO_2_ undergoes catastrophic growth under the same thermal and electrokinetic conditions. The decisive variable separating these two outcomes is therefore not the shared electrokinetic trend but the density-dependent hydrodynamic term. A complementary separation is provided by the base-fluid comparison: chemically identical CeO_2_ particles, exhibiting comparable temperature-induced reductions in zeta potential in both fuels, remain stable in the higher-viscosity n-dodecane (Test T12) yet fail in the lower-viscosity iso-octane (Test T03) at 80 °C. With surface chemistry held constant and only the base-fluid viscosity differing, this contrast isolates viscosity as the governing factor. Taken together, these two internal comparisons indicate that the temperature-dependent electrokinetic changes, while real, are not sufficient to account for the observed instability, and that the viscosity–density coupling remains the dominant driver—consistent with the central premise of the transport-physics framework.

#### 3.5.3 Electrokinetic Coupling and Qualitative Indicators

To reconcile the electrokinetic observations with the purely thermophysical theoretical framework, it is crucial to clarify the role of the Zeta potential (**ζ**) data. As stated in earlier sections, electrokinetic interactions (DLVO forces) were intentionally excluded from the derivation of the Stability Phase Maps to isolate the pure transport-driven physical ceiling (viscosity collapse). Therefore, the **ζ**-potential measurements presented here are not intended to define or alter the theoretical stability boundaries; rather, they serve strictly as qualitative indicators of the system’s actual physicochemical state under thermal stress. The experimental data (Table 5) show that for the unstable CeO_2_/iso-octane system at 80 °C, the measured Zeta potential dropped to −8.7 mV (Test T03), a trend consistent with a weakening of the Electrical Double Layer (EDL). At this point, however, a methodological caveat must be explicitly acknowledged. Electrophoretic light scattering, like DLS-derived sizing, fundamentally presupposes that particles undergo stable diffusive and electrophoretic motion within the suspension. In a system that is simultaneously aggregating and sedimenting (precisely the condition diagnosed for Test T03), this assumption is partially violated, and the reliability of the derived electrokinetic parameters is correspondingly reduced. Particle settling and the elevated effective size of agglomerates can distort the measured intensity and mobility,^81,82^ while multiple scattering in the destabilized dispersion further attenuates the recorded signal and biases the autocorrelation function.^83^ Consequently, the reported decrease in Zeta potential at elevated temperature should not be read as a precise quantification of surface charge, but as a semiquantitative indicator of a deteriorating electrokinetic state. A genuine reduction in Zeta potential magnitude with increasing temperature has also been independently reported for oxide nanoparticles,^84^ which suggests that the observed trend reflects a real, albeit imperfectly quantified, physicochemical change rather than a pure measurement artifact. The direction of any such artifact is itself a consequence of destabilization, since the loss of measurement fidelity arises only once the system has already begun to aggregate and settle. The electrokinetic data therefore do not define the stability boundary—a role reserved exclusively for the transport-physics model—but instead offer corroborating, qualitative evidence that the practical failure of the suspension coincides with the hydrodynamic ceiling predicted by the Lagrangian framework. In this sense, the simultaneous viscosity collapse and apparent EDL weakening together describe a coupled failure mode, in which the breakdown of the chemical barrier accelerates the system toward the physical limits identified in this study.

## 4 Conclusions

This study presents a predictive kinetic stability framework for hydrocarbon-based metal oxide nanofuels, delineating the often less explicitly addressed physical boundaries by isolating the thermophysical mechanisms governing sedimentation, independent of chemical stabilization (DLVO). The theoretical boundaries derived from the Lagrangian-based parametric modeling approach were experimentally corroborated as an idealized physical baseline, phenomenologically demonstrating that catastrophic macroscopic aggregation and settling occur exactly when this theoretical thermophysical ceiling is breached. The comprehensive analysis leads to the following fundamental conclusions:

- **Dominance of Viscosity-Induced Instability:** The study quantitatively identified the viscosity-temperature coupling as the primary driver of instability in high-temperature applications. Both analytical modeling and experimental results confirmed that a viscosity reduction of ∼55–65% (from 20 to 100 °C) eliminates the hydrodynamic drag necessary to suspend high-density particles. This degradation hydrodynamically suppresses the diffusive gain provided by the increase in thermal energy (∼1.27 times), driving the system into a gravity-dominated regime.
- **Experimental Confirmation of Phase Maps:** The proposed “stability phase maps” successfully predicted the density-dependent behavior of nanofuels under thermal stress. Experimental validation revealed that while low-density SiO_2_ (2.65 g cm^–3^) particles maintained remarkable stability across all temperatures (20–80 °C), high-density CeO_2_ (7.13 g cm^–3^) particles in iso-octane underwent catastrophic agglomeration at 80 °C, growing from ∼86 nm to ∼621 nm. This finding empirically validates the “steep instability gradients” predicted for heavy metal oxides in the theoretical maps.
- **Definition of a Theoretical Stability Ceiling:** A concrete engineering output of this study is the definition of a “first-order physical ceiling” for nanofuel design. For the iso-octane/CeO_2_ system, the maximum stable particle diameter was theoretically determined to decrease from ∼41 nm to ∼27 nm under engine conditions. The experimental failure of larger aggregates at 80 °C corroborates this limit, demonstrating that exceeding this physical ceiling renders chemical stabilization ineffective against the overwhelming gravitational forces.
- **The Critical Regime Transition (*Pe*** = ***Pe***_***crit***_ **):** Analyses revealed a strong correlation between the onset of instability and the system-specific critical Peclet contour (*Pe_crit_*) that corresponds to the practical 1 mm/day settling tolerance. It should be emphasized that, across the entire parameter space examined here, the computed Peclet numbers remain well below unity; the relevant transition is therefore not an absolute *Pe* ≈ 1 crossing but the breaching of this system-specific Pe_crit_ contour, which marks the point at which advective settling begins to dominate diffusive transport within the studied regime. The experimental observation that the measured reduction in zeta potential (to −8.7 mV) qualitatively accompanies this transition is consistent with a coupled thermophysical failure mode, although the electrokinetic data are interpreted only as a semiquantitative indicator of the destabilized state rather than as a defining stability criterion. This implies that keeping the system below this critical contour is a fundamental prerequisite for the efficacy of any subsequent surfactant treatment.

In summary, this work transforms the formulation of nanofuels from an empirical trial-and-error process into a rational design methodology grounded in transport physics. By providing a validated map of “safe operational zones,” this study offers a predictive tool to optimize particle size and fuel type selection prior to costly experimental synthesis. Ultimately, by establishing this first-order, idealized physical baseline, the present study provides the transport-physics reference required to rationally evaluate the limits of colloidal stability under severe thermal stress, while recognizing that morphology, surface functionalization, concentration, aggregation kinetics, and DLVO interactions will further modulate these boundaries in real systems.

## 5 Limitations and Future Research Directions

Although this study presents a comprehensive and experimentally validated thermophysical framework for determining the kinetic stability limits of nanofuels, it contains certain limitations inherent in the modeling approach and experimental scope adopted. To ensure scientific transparency and guide subsequent studies in the literature, these boundaries and future projections are critically evaluated as follows.

### 5.1 Limitations of the Study

- **Dilute Regime and Interparticle Interactions:** Both the theoretical model and the experimental validation were fundamentally considered under dilute assumptions (volume fraction ϕ ≪ 1%). This constraint was necessary to isolate single-particle settling mechanics from multiple scattering effects in DLS. However, in practical engineering applications, particles constantly interact via van der Waals attractions and electrostatic or steric repulsions. The current analytical framework intentionally neglects complex multibody aggregation kinetics to isolate first-order transport mechanisms. As correctly pointed out during the peer-review process, while the model utilizes a single-particle Lagrangian approach, the experimental reality involves dynamic nanoparticle (NP) aggregation. It is crucial to clarify that the proposed “stability phase maps” do not aim to simulate the growth kinetics of clusters; rather, they identify the “thermophysical trigger point.” Once the base fluid’s viscosity collapses beyond the predicted threshold, the hydrodynamic drag becomes insufficient to keep particles separated, thereby allowing van der Waals attractions to dominate and trigger the catastrophic aggregation observed in the present DLS measurements. Consequently, the model serves as an idealized physical baseline that predicts the onset of instability, while the experiments capture the resulting morphological evolution of the suspension. In physical systems exhibiting active particle–particle agglomeration, the actual stability boundaries will shift, causing the nanofuel suspension to fail at lower temperatures or smaller primary sizes than those predicted by this idealized baseline.
- **Surrogate vs Commercial Fuel Complexity:** To rigorously isolate the thermophysical variables (viscosity and density), high-purity analytical-grade surrogate fuels (iso-octane and n-dodecane) were utilized instead of commercial gasoline or diesel. Commercial fuels contain a complex matrix of additives (detergents, friction modifiers, and antifoaming agents) that could chemically alter the nanoparticle surface potential. While the physical instability mechanisms identified here (viscosity collapse) remain valid, the chemical interaction between commercial additives and nanoparticles represents a secondary variable that is not covered in this physical model.
- **Discrepancy in Particle Sizing:** A quantitative deviation exists between the primary crystallite size assumed in the analytical model (<25 nm, based on BET) and the hydrodynamic diameter measured in experiments (∼45–85 nm). This is a known phenomenon in colloidal science, as DLS measures the hydrodynamic radius, including the solvation shell and any soft agglomerates formed in the solvent, rather than the dry core size. Although this does not invalidate the observed relative instability trends, it highlights that the “effective” settling diameter in a fluid is inherently larger than the dry powder size. Therefore, the analytical model provides a robust theoretical limit, while actual macroscopic settling in complex solvated environments may manifest slightly earlier due to these solvation effects. It should further be acknowledged that dynamic light scattering, while well suited to resolving the onset of particle growth that accompanies destabilization, provides an indirect measure of macroscopic sedimentation, since aggregation and bulk settling are related but not strictly equivalent phenomena. Complementary techniques that probe settling more directly (such as multiple-light-scattering profiling (e.g., Turbiscan), optical transmission monitoring, or extended sedimentation-height and storage tests) would provide a more complete description of the long-term settling dynamics and represent a valuable direction for subsequent experimental work.
- **Reliability of Electrokinetic Measurements under Thermal Stress:** The zeta potential values reported in this study were employed solely as qualitative diagnostic indicators rather than as model inputs. It should nevertheless be acknowledged that electrophoretic and dynamic light scattering techniques presuppose stable diffusive and electrophoretic particle motion, an assumption that is partially compromised once the suspension begins to aggregate and settle under thermal stress. Under these conditions, the quantitative accuracy of the derived electrokinetic parameters is inherently reduced, and the corresponding values (particularly at the highest temperature investigated) are better interpreted as semiquantitative trends than as precise measurements. This limitation does not affect the central conclusions of this study, since the predictive stability boundaries are defined entirely by transport-physics parameters (viscosity and density) and remain independent of the electrokinetic data.
- **Scope of Experimental Validation Across Material Diversity:** The analytical framework spans ten metal oxides, whereas the experimental validation employs three representative materials (CeO_2_, Fe_2_O_3_, SiO_2_). These were deliberately selected to span the full density range of the modeled oxides (2.65–7.13 g cm^–3^), since density is the first-order parameter governing the gravitational term in the transport model, and they accordingly provide a robust test of the density-dependent behavior predicted by the Phase Maps. It should nonetheless be acknowledged that three oxides cannot reproduce the full diversity of surface chemistry, crystallographic structure, and interfacial properties present across the ten modeled materials, all of which modulate the aggregation kinetics that drive a suspension toward the predicted hydrodynamic limit. The experimental results should therefore be regarded as validating the first-order, density-governed transport behavior of the framework rather than as establishing its quantitative universality across every oxide chemistry. Broader validation encompassing a wider range of surface chemistries represents a natural extension of this work.

Furthermore, the current methodology relies on a one-way coupled single-particle approach, consciously omitting two-way fluid-particle and particle–particle momentum exchanges. While incorporating generalized Boussinesq-Basset-Oseen (BBO) history forces or fully coupled transient aggregation kinetics would theoretically enhance the resolution of the model, it would also introduce severe computational overhead and intractable numerical stiffness without fundamentally altering the primary thermophysical trigger point identified herein.

### 5.2 Future Research Directions

The “stability phase maps” introduced in this study constitute a robust physical baseline for constructing more complex multiphysics models. The following extensions are recommended for future research:

- **Integration of Non-Newtonian Rheology:** Future models should incorporate concentration-dependent viscosity correlations (e.g., Krieger–Dougherty or Ostwald-de Waele models) to predict stability boundaries in the nondilute regime, where rheological properties become a function of shear rate and particle loading.
- **Combustion and Performance Testing**: With the physical stability limits now clearly defined and experimentally verified, the next logical step is to evaluate the combustion characteristics (ignition delay, flame speed, and microexplosion phenomena) of these optimized nanofuels in a reacting spray environment.
- **Hybrid Stability Maps (DLVO-Coupled):** Future studies could integrate the physical “phase maps” with chemical DLVO potentials. By superimposing the viscosity-induced instability zones defined in this study with electrostatic repulsion barriers, a unified “thermo-chemical stability map” could be generated to optimize both surfactant selection and particle size simultaneously.

Building upon the insights gained from this study, future research should focus on coupling the present quasi-steady Lagrangian framework with Population Balance Equations (PBE) or Discrete Element Method (DEM) approaches. Integrating temperature-dependent DLVO interactions and dynamic aggregation kinetics into the current model would transition this “idealized physical baseline” into a fully predictive computational tool capable of simulating real-world multiphase agglomeration and transient cluster growth under severe thermal stress.

## Supplementary Material



## Data Availability

The complete data set generated in this study is provided as Supporting Information accompanying this article. The thermophysical property data used as model inputs are available from the literature sources cited within the manuscript.

## Notes

Complete parametric data set (data set.csv): particle name; base fluid; temperature (°C); fuel density (kg/m3); fuel viscosity (Pa·s); particle diameter (nm); settling velocity (m/s); Brownian diffusion coefficient (m2 /s); Peclet number; settling rate (mm/day) — for all ten metal oxide nanoparticles in both base fluids (iso-octane and n-dodecane) across the full temperature (20–100 °C) and particle-diameter (10–100 nm) range, N = 15,640 data points (CSV)
